# Effectiveness of two-stage revision with commercial polymethylmethacrylate articulated hip spacer: similar outcomes against monomicrobial and polymicrobial hip periprosthetic joint infections

**DOI:** 10.5194/jbji-8-51-2023

**Published:** 2023-02-06

**Authors:** Leonel Perez Alamino, German Garabano, Joaquín Anibal Rodriguez, Matías Cullari, Hernán Del Sel, Cesar Angel Pesciallo

**Affiliations:** Department of Orthopaedic and Traumatology, British Hospital of Buenos Aires, Perdriel 74 C1280 AEB, Argentina

## Abstract

**Background**: orthopaedic surgeons still struggle against a devastating complication – periprosthetic joint infection (PJI). A two-stage revision is considered the gold standard for chronic PJI for several authors, with success rates over 90 %. This strategy implies the remotion of the prosthesis and the implantation of an antibiotic-impregnated cement spacer in the joint. The primary objective of this study was to assess the effectiveness of a two-stage revision approach using a commercial prefabricated antibiotic-impregnated cement hip spacer for the treatment of hip PJI regarding monomicrobial and polymicrobial infections. Secondly, to assess risk factors for failure of two-stage revision. **Material and methods**: we conducted a retrospective study on patients that underwent revision of total hip arthroplasty (THA) between January 2002 and January 20218. We included adult patients with a diagnosis of chronic hip PJI that underwent two-stage revision using a prefabricated gentamicin-impregnated cement of polymethylmethacrylate (PMMA) hip spacer. We assessed whether it was monomicrobial or polymicrobial infections and comorbidities. Treatment success was defined when eradication of the infection was observed and no further procedures or mortality were registered after the second stage. Persistence or recurrence of infection was considered a failure of treatment. **Results**: the final series consisted of 84 patients treated with the same hip spacer: 60 (71.4 %) monomicrobial and 24 (28.6 %) polymicrobial joint infections with an overall follow-up of 59.0 (36.0–84.0) months. The overall success rate was 90.5 %. Eight (9.5 %) patients failed. Smoking and BMI greater than 30 m kg ${}^{-2}$ were identified independent risk factors for failure in multivariate analysis. **Conclusion**: our study suggests that prefabricated gentamicin-impregnated PMMA spacer is an effective tool for the treatment of PJI, achieving similar outcomes whether it is monomicrobial or polymicrobial infections. Randomized prospective studies are needed to obtain more reliable conclusions.

## 1 Introduction

Despite the excellent long-term results reported about total hip replacements regarding joint osteoarthritis (Synder et al., 2012; Chang and Haddad, 2020; Lucchini et al., 2021), orthopedic surgeons continue to struggle against a devastating complication: periprosthetic joint infection (PJI) (Kapadia et al., 2015). In primary arthroplasties, their incidence is about 0.5–3 %, but in hip revision, this number increases to 14.8 %. (Phillips et al., 2006; Leone et al., 2010; Parvizi et al., 2010).

Several authors consider two-stage revision to be the gold standard for chronic PJI, with success rates exceeding 90 % (Amanatullah et al., 2018; Pignatti et al., 2010; Younger et al., 1998, 1997). This strategy involves the removal of all prosthesis components, surgical debridement, irrigation, and implantation of an antibiotic-impregnated cement spacer. (Hsieh et al., 2004). The benefit focuses on occupying a “dead space” and being able to release high local concentrations of antibiotics into the joint environment (Grassi et al., 2007).

There are several types of hip spacer designs, and according to their manufacture we can mention those handcrafted in the operating room and those commercially available (Veltman et al., 2016). Among the advantages of the latter spacers is that their use shortens surgical times, and both types allow for maintaining joint mobility, the absence of soft tissue contraction, and ensures easier reimplantation (Konstantinos et al., 2006).

Since the 2000s, prefabricated antibiotic-impregnated cement spacers (Subiton^®^; Laboratorios SL, BA, Argentina) have been used in our field for the treatment of chronic PJI, and to our knowledge, there are no studies about their outcomes that compare when treating infections with an isolated and with multiple microorganisms.

The primary objective of this study was to assess the efficacy of a two-stage revision approach using a commercial prefabricated antibiotic-impregnated cement hip spacer for the treatment of hip PJI regarding monomicrobial and polymicrobial infections. Secondly, to evaluate risk factors for failure of the two-stage revision.

## 2 Material and methods

With the Institutional Review Board`s approval, we conducted a retrospective study of patients undergoing revision of THA between January 2002 and January 2018.

Inclusion criteria for the study were adult patients ( >18 years) with a diagnosis of chronic hip PJI ( >4 weeks of primary THA) who underwent a two-stage revision using a prefabricated gentamicin-impregnated cement of polymethylmethacrylate (PMMA) hip spacer (Subiton^®^, Laboratorios SL, Buenos Aires, Argentina) and completed a minimum follow-up of 3 years after the second stage.

Patients with previous hip revision, infections with negative culture, and those who did not complete the two stages of revision were excluded from the study.

PJI was defined according to the 2018 Musculoskeletal Infection Society (MSIS) criteria (Parvizi and Gehrke, 2018). Patient medical records were reviewed, and the following information was registered: gender, age, body mass index (BMI), Charlson comorbidity index (CCI), smoking (patients that were smokers at the time of the first stage), involved microorganisms, length of hospital stay, antibiotic therapy duration, and follow-up. We also documented serum levels of erythrocyte sedimentation rate (ESR), C-reactive protein (CRP), and white blood cell (WBC) count before the first and second stages.

In the first stage approach, there was a period of 10–14 d in which broad-spectrum intravenous antibiotic therapy was administered postoperatively. After this, it was continued with selective oral therapy that was adjusted according to the resistance profile of the infecting microorganism, guided by the infectious disease department.

### 2.1 Surgical technique

The first stage consisted of surgical debridement of devitalized tissue and removal of both components of the prosthesis. A minimum of five samples were sent to bacteriology for analysis. Later, a gentamicin-impregnated (2.5 g) hip spacer was implanted. To avoid implant rotation, the space between the implant and the proximal femur was filled with antibiotic cement (vancomycin 1 g, per dose of cement).

After completing systemic antibiotic therapy, patients underwent a minimum of 2-week “antibiotic holiday” before to the second stage (Restrepo et al., 2014). Reimplantation was decided in conjunction with the department of infectious diseases when a decrease in serum biomarkers and absence of pain was observed.

### 2.2 Rehabilitation protocol

All patients underwent the same rehabilitation protocol. After the first stage, they were allowed to walk on the first day after surgery with the aid of a walker or two canes, and were encouraged to restrict hip flexion above 90 ${}^{\circ }$ and maximum range of rotation.

Between the first and second stages, a physical examination, a visit with an infectious disease specialist, and laboratory tests were scheduled every 2 weeks.

Clinical and functional outcomes were assessed by comparing the values of two scores registered annually at each routine patient visit: modified Harris hip score (mHHS) and WOMAC (Western Ontario and McMaster Universities Osteoarthritis Index) (Harris, 1969; Bellamy et al., 1998).

Persistence (same microorganism as original infection) or recurrence (new infection) was considered a treatment failure of the two-stage revision (Palmer et al., 2020). Treatment success for this study was considered when eradication of the infection was observed, and no further procedures or mortality were registered after the second stage (Delphi Multidisciplinary International Consensus) (Diaz-Ledesma et al., 2013).

## 3 Statistical analysis

A normal distribution test was performed. Continuous variables were described as mean and standard deviation or median and interquartile range (according to normality) and categorical variables as frequency and percentage. Comparison of clinical and functional outcomes were assessed with the Student's t test and categorical variables were analyzed with the chi-square ( $X^{2}$ ) test (or Fisher's exact test if needed).

To calculate risk factors, continuous variables were transformed into categorical ones as follows: age – <70 and >70 years old, and BMI – 20–24, 24–30, and >30 kg m ${}^{-2}$ . After this, univariate logistic regression was calculated to identify potential risk factors for failure. A difference of p<0.05 was considered statistically significant. All the data were collected into an Excel^®^ (Microsoft, Redmon, USA) spreadsheet, and statistical calculations were performed with the software GraphPad Prism 8.0^®^ (LaJoya, CA, USA).

## 4 Results

### 4.1 Study population and patient's characteristics

During this period, 93 hip PJI underwent two-stage revisions: six (6.4 %) of these were cases of infection with negative cultures, one (1.1 %) did not complete minimum follow-up, and two (2.1 %) did not complete the two stages, since their comorbidities did not make them candidates for another surgery, so they were excluded.

The final series consisted of 84 patients: 60 (71.4 %) monomicrobial and 24 (28.6 %) polymicrobial joint infections with an overall follow-up of 59.0 (36.0–84.0) months.

Characteristics of both cohorts and microorganisms involved are described in Tables 1 and 2.

**Table 1:** Preoperative characteristics of patients included in the study.

Variables	Monomicrobial ( n=60 )	Polymicrobial ( n=24 )	P value
Age (median, IQR)	70.5 (63.2–79.0)	64.0 (56.0–70.0)	<0.01
Sex ( n,% )			
Male	34 (56.7)	12 (50.0)	0.57
Female	26 (43.3)	12 (50.0)	
BMI (median, IQR)	28.1 (26.3–29.9)	26.4 (25.7–30.3)	0.27
Smoking	13 (21.7)	8 (33.3)	0.26
Charlson comorbidity index ( n,% )			
III	29 (48.3)	12 (50.0)	0.99
IV	18 (30.0)	8 (33.3)	0.79
V	1 (1.7)	2 (8.3)	0.21
VI	11 (18.3)	1 (4.2)	0.99
VII	1 (1.7)	1 (4.2)	0.49
Preoperative mHHS (median, IQR)	52.4 (49.1–55.1)	54.5 (49.6–56.8)	0.23
Preoperative WOMAC (median, IQR)	39.3 (37.2–43.4)	40.8 (37.5–45.6)	0.12

IQR: interquartile range. BMI: body-mass index. mHHS: modified Harris hip score. WOMAC: Western Ontario and McMaster Universities Osteoarthritis Index.

**Figure 1:**
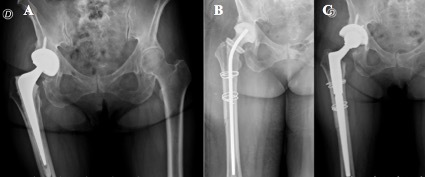
**(a)** Hybrid total hip replacement (THR) with demarcation around femoral stem. **(b)** Long antibiotic-impregnated cement spacer. **(c)** Femoral reconstruction with distal fixation stem 14 weeks after first-stage revision.

The median time between primary arthroplasty and the first stage was 9.0 (range 6.0–12.5) and 8.0 (range 5.0–14.0) weeks regarding monomicrobial and polymicrobial PJI ( p=0.10 ), respectively. The duration of systemic antibiotic therapy was 14.5 weeks (range 14.0–42.0) for monomicrobial infections and 16.0 weeks (range 14.0–46.0) for polymicrobial infections ( p=0.8 5). The IV antibiotic treatment was continued by a median 4 weeks (range 2–14). No mechanical complications associated with spacers like pain, fracture, or dislocation were reported (Fig. 1).

**Table 2:** Microorganisms identified in hip PJI of the series.

Microorganism	Monomicrobial	Polymicrobial
	( n=60 )	( n=28 )
*S. aureus*	31 (51.7)	2 (8.3)
CoNS	18 (30.0)	4 (16.7)
Enterococcus	3 (5.0)	9 (37.5)
*Klebsiella pneumoniae*	3 (5.0)	3 (12.5)
*Escherichia coli*	2 (3.3)	1 (4.2)
Bacteroides spp.	0 (0)	3 (12.5)
Propinebacterium	2 (3.3)	0 (0)
*Pseudomona aeuruginosa*	0 (0)	2 (8.3)
*Candida albicans*	1 (1.7)	0 (0)

CoNS: coagulase negative Staphylococcus. *S. aureus*: *Staphylococcus aureus*.

In 45 (54.8 %) of the infections, microorganisms sensitive to gentamicin were identified, while in 38 (41.7 %) cases, the microorganisms were sensitive to vancomycin. The patient who developed a fungal infection was treated with caspofungin.

The settings of the patients included in the study are described in Table 3.

**Table 3:** Postoperative data summary of patients with monomicrobial and polymicrobial hip PJI.

	Monomicrobial ( n=60 )	Polymicrobial ( n=24 )	P value
First stage			
Hospital stay (d) ${}_{\left(\mathrm{median},\;\mathrm{IQR}\right)}$	9.0 (8.0–10.0)	9.5 (6.5–12.5)	0.13
ESR ${}_{\left(\mathrm{median},\;\mathrm{IQR}\right)}$	44.5 (32.5–60.0)	47.5 (19.0–80.7)	0.59
CRP ${}_{\left(\mathrm{median},\;\mathrm{IQR}\right)}$	12.0 (4.7–34.2)	24.0 (23.5–37.0)	<0.01
WBC ${}_{\left(\mathrm{median},\;\mathrm{IQR}\right)}$	8900 (7525–10750)	10 650 (9150–13875)	<0.01
Second stage			
Hospital stay (d) ${}_{\left(\mathrm{median},\;\mathrm{IQR}\right)}$	7.0 (5.0–8.0)	8.0 (5.0–8.0)	0.14
ESR ${}_{\left(\mathrm{median},\;\mathrm{IQR}\right)}$	15.0 (10.25–21.0)	16.0 (11.0–28.0)	0.11
CRP ${}_{\left(\mathrm{median},\;\mathrm{IQR}\right)}$	1.0 (0.7–2.0)	1.5 (0.6–2.3)	0.04
WBC ${}_{\left(\mathrm{median},\;\mathrm{IQR}\right)}$	6700 (4800–8175)	8750 (8100–11 600)	<0.01
Failure ${}_{\left(n,\;\%\right)}$	5 (8.3)	3 (12.5)	0.68
Follow-up (months) ${}_{\left(\mathrm{median},\;\mathrm{IQR}\right)}$	38.0 (36.0–44.0)	41.0 (38.0–84.1)	0.12

IQR: interquartile range. ESR: erythrocyte sedimentation rate. CRP: C-reactive protein. WBC: white blood cell.

The reimplantation was performed at a median of 16.0 (14.0–27.0) weeks.

We observed statistically significant improvement regarding mHHS and WOMAC scores in both cohorts (Table 4). When comparing the values of mHHS and WOMAC after the second stage, we found no significant differences between the patients with monomicrobial and polymicrobial joint infections (mHHS: 84.2 versus 83.3; p=0.68 and WOMAC: 82.8 versus 84.2; p=0.47 ).

**Table 4:** Score values before first stage and after second stage of hip revision.

Scores	Before first stage	After second stage	P value
mHHS			
Monomicrobial	52.4 (49.1–55.1)	84.2 (73.7–89.6)	<0.01
Polymicrobial	54.5 (49.6–56.8)	83.3 (78.5–88.4)	<0.01
WOMAC			
Monomicrobial	39.3 (37.2–43.4)	82.8 (79.2–87.7)	<0.01
Polymicrobial	40.8 (37.5–45.6)	84.2 (73.7–89.6)	<0.01

HHS: modified Harris hip score. WOMAC: Western Ontario and McMaster Universities Osteoarthritis Index.

**Table 5:** Comparison of patients with and without failure.

Variable	Failure ( n=8 )	Non-failure ( n-76 )	P value
Age	68.0 (51.5–75.5)	71.0 (65.0–78.0)	0.30
Male	5 (62.5)	31 (40.8)	0.27
BMI	30.4 (26.4–32.0)	28.1 (24.3–30.2)	0.03
Smoking	6 (75)	25 (32.9)	0.04
CCI			
III	0 (0)	41 (53.9)	<0.01
IV	3 (37.5)	23 (30.2)	0.69
V	2 (25.0)	1 (1.3)	0.08
VI	2 (25.0)	10 (13.1)	0.10
VII	1 (12.5)	1 (1.3)	0.18
Type of infection			
Monomicrobial	5.0 (62.5)	55.0 (72.4)	0.68
Polymicrobial	3.0 (37.5)	21.0 (27.6)	

### 4.2 Success and failure rate

The overall success rate was 90.5 %. Eight (9.5 %) patients failed. Five (62.5 %) were patients with an isolated microorganism and three (37.5 %) were polymicrobial joint infections. Though the latter group had a higher percentage of failure, this difference was not statistically significant (8.3 % versus 12.5 %; p=0.6 8).

After the second stage, four of the patients with monomicrobial infections and two of the polymicrobial group required surgical debridement, irrigation, and specific antibiotic therapy at a median of 3.0 (2.0–5.0) weeks. The two remaining patients underwent a new revision with an antibiotic-impregnated cement spacer (Table 5).

### 4.3 Failure risk factors

We observed significant association between smoking and BMI greater than 30 m kg ${}^{-2}$ and failure (Table 6).

**Table 6:** Univariate logistic regression of risk factors for failure.

Variables	OR (CI95 %)	P value
Age <70 or >70	0.54 (0.13–2.29)	0.47
Sex	0.26 (0.05–1.15)	0.18
CCI <4 or >4	1.05 (0.28–3.84)	0.99
Smoking	14.10 (2.88–71.20)	<0.01
BMI <30 or >30 m kg ${}^{-2}$	14.49 (2.92–71.93)	<0.01

OR: odds ratio. CI95 %: confidence interval 95 %. CCI: Charlson comorbidity index. BMI: body mass index. Multivariate analysis identified smoking and BMI >30 m kg ${}^{-2}$ as independent risk factors for failure.

**Table 7:** Multivariate analysis of risk factors for failure.

Variables	OR (IC95 %)	P value
Smoking	12.94 (3.17–60.41)	<0.01
BMI <30 or >30 m kg ${}^{-2}$	2.14 (1.32–9.22)	<0.01

OR: odds ratio. CI95 %: confidence interval 95 %. BMI: body mass index.

## 5 Discussion

Prefabricated antibiotic PMMA hip spacers have initially been impregnated with aminoglycosides, but recent designs have allowed the addition of vancomycin. Although they traditionally contain low doses of antibiotic, they can achieve higher local concentrations because of their dimpled surface (Scott, 2020).

The main finding of our study is that we observed that the use of prefabricated antibiotic-impregnated cement spacers is an effective tool for the treatment of PJI, with a success rate of 90.5 % and with no significant differences regarding functional outcomes between patients with monomicrobial or polymicrobial infections.

Although we have not found previous series reporting the use of the spacers used in the present study, comparatively, our success rate was as reported by Hsieh et al. (2004). These authors assessed 40 patients with a recurrence rate of 2.5 % with 4 years of follow-up. Likewise, Romanó et al. (2011) in a prospective cohort study of 20 patients who underwent a two-stage hip revision using antibiotic-impregnated articulating hip spacers reported a 95 % success rate.

Polymicrobial hip PJI has been traditionally considered a risk factor for failure in hip revision (Della Valle, 2011), and its incidence is between 6 and 37 % (Pulido et al., 2008; Holleyman et al., 2016; Moran et al., 2007; Peel et al., 2012). In our series, both cohorts achieved promising success at the end of the study, and though we observed a higher percentage of failure (12.5 %) in the polymicrobial group, the difference versus patients with monomicrobial (8.3 %) joint infections was not statistically significant. These outcomes agree with the findings of Bozhkova et al. (2016): they reported a higher failure rate in patients with polymicrobial infections (34.1 %) compared to the monomicrobial cohort (23.4 %) after two-stage revision, but this was not statistically significant. Another finding is that regardless of whether it was monomicrobial or polymicrobial PJI, both achieved statistical improvements after second-stage surgery regarding WOMAC and mHHS scores, with no significant differences between them.

Previously, other studies have described similar findings: Pignatti et al. (2010) reported values greater than 80 after 5.3 years of follow-up, analyzing 60 patients that underwent a two-stage hip revision for PJI treatment.

In addition, significantly higher levels of CRP and WBC were observed in the polymicrobial group when comparing monomicrobial infections after the second stage, but within normal ranges. Bozhkova et al. (2016) also reported higher percentages of CRP levels in patients with polymicrobial (42.6 %) joint infections with failure of second stage, though this was not significant. Similarly, Mortazavi et al. (2011) analyzed 117 patients that underwent two-stage revision for PJI from a prospective database and failed to find an association between CRP and ESR levels with failure. The authors of this study agree that there are no clear cut-off values of these biomarkers to predict failure. We consider every patient as a singular case, searching for a combination of laboratory, radiographic, and clinical parameters to decide on reimplantation.

Logroscino et al. (2019) reported that patients with BMI >25 had significant association with reinfection after two-stage hip revision. Likewise, Jhan et al. (2017) assessed 61 cases of hip PJI that underwent two-stage revision and observed that BMI >30 kg m ${}^{-2}$ was associated with higher rates of failure.

In concordance with these authors, uni- and multivariate analysis showed a statistically significant association between failure and BMI >30 kg m ${}^{-2}$ in our study.

Furthermore, this study found that smoking patients were 12.94 times (CI95 % 3.17–60.41) more likely to fail after reimplantation than non-smokers. This agrees with Ahmad et al. (2019), who found a 3.9 (CI95 %1.1–14.6i) increased risk of failure in smokers after analyzing 97 patients undergoing two-stage revision for PJI.

In addition, the group of Parvizi (Aali Rezaie et al., 2018) described a significant association between greater CCI and failure after reimplantation in regression analysis with an odds ratio of 1.40 (CI95 % 1.06–1.86). Although our findings do not agree with the latter, we must be careful, because there is a trend to believe that the greater the comorbidities of the patients, the greater the rate of failure.

Our study is not without limitations: we must mention that it is a retrospective study, and we did not consider every possible preoperative comorbidity, which might represent a confounding factor. On the other hand, this paper analyzed an adequate number of patients that were operated on in the same institution by the same trained hip-revision surgeons. We have the task left to continue with long-term follow-up and to establish a control group to give greater relevance to our findings.

## 6 Conclusions

Our study suggests that gentamicin-impregnated PMMA hip spacer is an effective tool for the treatment of PJI, achieving similar outcomes whether it is monomicrobial or polymicrobial infections. Randomized prospective studies are needed to obtain more reliable conclusions.

## Data Availability

All data generated and analyzed during this study are included in this published article and are available from the corresponding author on reasonable request.
